# Importance and Involvement of Imidazole Structure in Current and Future Therapy

**DOI:** 10.3390/molecules31030423

**Published:** 2026-01-26

**Authors:** Alexandra Pavel Burlacu, Maria Drăgan, Ovidiu Oniga, Mădălina Nicoleta Matei, Ilioara Oniga, Elena-Lăcrămioara Lisă, Claudia-Simona Stefan, Oana-Maria Dragostin

**Affiliations:** 1 Research Centre in the Medical-Pharmaceutical Field, Faculty of Medicine and Pharmacy, “Dunarea de Jos” University of Galati, 35 AL Cuza Street, 800010 Galati, Romania; alexandra.burlacu@ugal.ro (A.P.B.); madalina.matei@ugal.ro (M.N.M.); elena.lisa@ugal.ro (E.-L.L.); claudia.stefan@ugal.ro (C.-S.S.); 2 Department of II Pharmaceutical Science, Faculty of Pharmacy, Grigore T. Popa University of Medicine and Pharmacy Iasi, 700115 Iași, Romania; maria.wolszleger@umfiasi.ro; 3 Department of Pharmaceutical Chemistry, “Iuliu Hațieganu” University of Medicine and Pharmacy, 41 Victor Babeș Street, 400012 Cluj-Napoca, Romania; 4 Department of Pharmacognosy, Faculty of Pharmacy, “Iuliu Hațieganu” University of Medicine and Pharmacy, Ion Creangă Street 12, 400010 Cluj-Napoca, Romania; ioniga@umfcluj.ro

**Keywords:** imidazole, heterocyclic drugs, diazoles, biological compounds

## Abstract

Imidazole is, from a structural point of view, a heterocycle consisting of three C atoms and two N atoms, belonging to the class of diazoles, having two N atoms at the first and third positions in the aromatic ring. Being a polar and ionizable aromatic compound, it has the role of improving the pharmacological properties of lead molecules, thus being used to optimize their solubility and bioavailability. Imidazole is a constituent of many important biological compounds, like histidine, histamine, and purine compounds, the most widespread heterocyclic compound in nature. In current practice, substituted imidazole derivatives play a major role in antifungal, antibacterial, anti-inflammatory, CNS active compounds, antiprotozoal, as well as anticancer therapy. Thus, imidazole derivatives have demonstrated significant anticancer activities by inhibiting the key metabolic pathways essential for tumor cell growth and survival. Nitroimidazoles, for instance, have been employed as hypoxia-directed therapeutic agents, targeting oxygen-deprived tumor tissues, while mercaptopurine derivatives are well-established in oncological treatments. Structural modifications of the imidazole nucleus have led to the novel compounds exhibiting increased selective cytotoxicity against cancer cells, while sparing normal healthy cells. In accordance with what has been stated, this review highlights recent research on the medicinal and pharmaceutical interest of novel imidazole derivatives, emphasizing their potential in the development of new drugs.

## 1. Introduction

Heterocyclic chemistry has long been a cornerstone of pharmaceutical innovation, with nitrogen-containing rings playing a central role in the development of bioactive molecules. Among these, the imidazole ring stands out as a privileged structure in medicinal chemistry, owing to its unique electronic configuration, aromatic character, and ability to engage in a wide variety of chemical interactions.

The imidazole nucleus plays an essential role in medicinal chemistry, due to its derivatives which have demonstrated significant biological activity; therefore, imidazole and its derivatives have garnered significant attention in recent years due to their broad pharmacological potential and structural versatility [1,2].

Imidazole is the main structure of many well-known compounds of the human organism, such as histidine, histamine, vitamin B12, biotin, and purines, which is formed by fusion with the pyrimidine nucleus [3,4,5,6,7,8]. At the same time, it is a component of many molecules with therapeutic effect, both natural and synthetic, such as cimetidine, azomycin, delamanid, and metronidazole. More recently, derivatives with various biological activities and pharmacological properties, including antimicrobial, antiviral, antifungal, anti-inflammatory, and anticancer effects, were obtained by substitution of the imidazole nucleus [9,10,11,12,13,14,15,16,17,18,19,20,21,22,23].

From a structural perspective, imidazole is a planar, five-membered heterocyclic ring [24] composed of three carbon atoms and two nitrogen atoms, with the nitrogen atoms at the first and third positions, which places imidazole within the class of diazoles [25,26,27].

**Figure 1:**
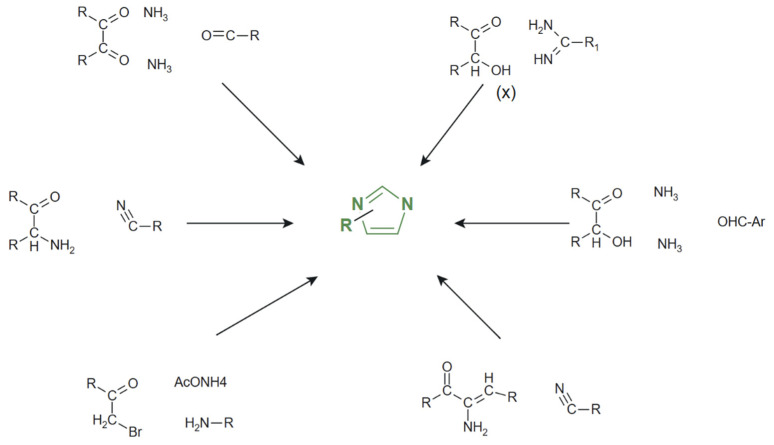
Main synthetic approaches to imidazoles [28].

The compound was originally named glyoxaline by Heinrich Debus, based on its initial synthesis from glyoxal and formaldehyde in an ammonia medium. Figure 1 illustrates the evolution of imidazole chemistry, highlighting not only the classical Debus synthesis but several modern synthetic routes for the preparation of substituted imidazole derivatives [28,29,30].

In general, most of the reported studies describe the formation of biologically active imidazole derivatives through single-step synthetic protocols, typically affording the target products in yields exceeding 70%. Such one-step approaches highlight the efficiency and practicality of these methods for medicinal chemistry applications. Moreover, some protocols, including those employing ethylene glycol as the reaction medium, use relatively sustainable solvents and mild reaction conditions, in line with green chemistry principles. However, despite these advantages, scalability remains limited, as most studies report product isolation at small laboratory scales and often rely on classical condensation reactions, organic solvents, and chromatographic purification. While suitable for laboratory-scale evaluation, such approaches present limitations also in terms of sustainability and waste generation, underscoring the need for greener alternatives when potential therapeutic candidates advance toward larger-scale development.

Imidazole is a highly polar compound, with a calculated dipole moment of approximately 3.61 Debye [29,30,31,32]. Its aromaticity is attributed to a sextet of π-electrons, comprising a lone pair from the protonated nitrogen atom and one π-electron from each of the other four ring atoms. Its amphoteric nature allows it to function both as a weak acid and as a weak base under physiological conditions [33].

Given the ongoing global demand for more effective and selective therapeutic agents, the exploration of imidazole chemistry continues to be a dynamic and promising field. The unique physicochemical properties and biological relevance of the imidazole nucleus have prompted us to explore a wide range of imidazole derivatives with diverse pharmacological applications. Thus, through this work, we aim to identify and analyze the structural modifications at various positions on the imidazole ring [34,35,36,37] which have led to new compounds with improved therapeutic profiles and reduced toxicity.

## 2. The Biological Activities of Imidazole Derivatives

According to a substantial body of evidence, imidazole derivatives constitute a highly versatile class of heterocyclic compounds with extensive pharmacological relevance, owing to their ability to modulate diverse biochemical targets. The biological profile of imidazole-based compounds is profoundly influenced by the nature, position, and electronic properties of substituents on the imidazole ring or adjacent scaffolds, which modulate target binding, selectivity, and bioavailability.

Accordingly, some molecules have consistently demonstrated significant antifungal and antibacterial activity, primarily through the inhibition of cytochrome P450-dependent enzymes involved in ergosterol biosynthesis, which leads to the accumulation of 14α-methyl sterols, the depletion of ergosterol in the fungal membrane, the disruption of membrane integrity and permeability, and ultimately to the inhibition of fungal growth and replication [38,39,40,41,42].

In parallel, several imidazole-based agents have exhibited potent anti-inflammatory properties, often linked to the suppression of pro-inflammatory mediators such as prostaglandins and cytokines [43,44,45,46,47,48]. Their antitubercular potential has been particularly notable in the context of multidrug-resistant *M. tuberculosis* strains, with compounds like Delamanid and Pretomanid illustrating clinical utility [49,50,51]. Imidazole scaffolds also contribute to gastroprotective mechanisms via H2 receptor antagonism and proton pump inhibition, supporting their anti-ulcer efficacy [52,53]. Similarly, in the metabolic domain, imidazole derivatives have shown promising antihyperglycemic effects through the inhibition of α-glucosidase and α-amylase enzymes, thereby modulating postprandial hyperglycemia [54]. A distinct subgroup of imidazole derivatives has shown remarkable antiparasitic efficacy in human medicine, exemplified by clinically established 5-nitroimidazoles, such as metronidazole, tinidazole, and ornidazole, which act through reductive bioactivation under anaerobic conditions to target protozoan pathogens, including *G. lamblia*, *T. vaginalis*, and *E. histolytica* [55]. In addition, several imidazole derivatives have exhibited preferential antiproliferative activity toward specific cancer cell lines, such as MCF-7 (breast cancer) and HT-29 (colorectal cancer), a trend that has been consistently reported by Vasamsetti et al. [56], Alzahrani et al. [57], Al-Qahtani et al. [58], Chen et al. [59], and others. These studies have attributed the selective anticancer effects of imidazole-based compounds to mechanism-driven processes, including dual pathways involving p53 induction and microtubule inhibition, as well as alkylating activity or inhibition of DNA synthesis.

Notably, the preferential activity observed for certain cancer cell lines suggests that imidazole derivatives may exploit tumor-specific vulnerabilities, rather than act as nonspecific cytotoxic agents. In particular, mechanisms such as microtubule disruption and p53 pathway activation are more pronounced in rapidly proliferating and genetically unstable cancer cells, which may explain the cancer type-dependent responses reported in these studies [60,61].

When these mechanisms are considered in the context of the hallmark features of cancer cells—such as accelerated cell division, genomic instability, and frequent DNA replication—they provide a rational explanation for the enhanced susceptibility of tumor cells compared to normal cells.

Additional support for this hypothesis is provided by independent literature reports, including studies by Nasrollahzadeh et al. [62] or Golcienė et al. [60] and Amirmostofian et al. [55], in which imidazole-based derivatives were evaluated on both tumor and normal cell models, revealing preferential cytotoxicity toward malignant cells, despite moderate or limited effects on normal cell viability [62,63].

Collectively, these multifaceted activities underscore the therapeutic potential of imidazole-based compounds across a wide range of pathological conditions.

### 2.1. Antifungal Activity

Imidazole derivatives act on the ergosterol synthesis pathway by blocking the activity of lanosterol 14-alpha-demethylase, determining the modification of the structure and permeability of the fungal membrane by selectively inhibiting cytochrome P450-dependent lanosterol demethylation [38]. The first azole compound with antifungal activity was reported by Woolley in 1944 [39], who, for the first time, declared the antifungal effect of the benzimidazole fragment; but, the first drug, clotrimazole, was developed only in 1958 [40,41,42].

Ketoconazole and other imidazole derivatives, such as miconazole, econazole, and itraconazole, have been used as effective therapeutic agents against mycotic infections. Ketoconazole inhibits the P-450 enzyme-dependent biosynthesis of ergosterol from lanosterol in fungi, leading to their eventual destruction [42].

Antifungal imidazoles constitute an important and continuously expanding class [57,58,59,60,61] and currently, they are used in a variety of pharmaceutical forms (lotions, tablets, creams, etc.) in relatively low concentrations of 1–2% [64,65,66,67,68].

**Figure 2:**
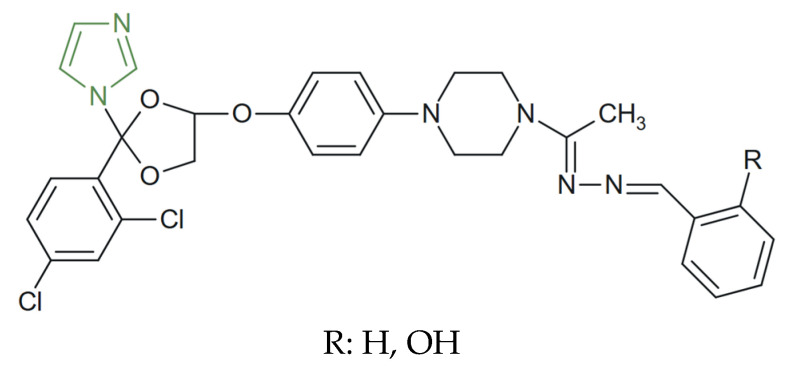
Structural representation of novel ketoconazole derivatives [68].

This class of compounds is under continuous development. Notably, Lisa et al. synthesized and characterized new imidazole derivatives with an activity spectrum similar to that of ketoconazole. These compounds (Figure 2) were encapsulated in β-cyclodextrin complexes to improve bioavailability and physicochemical stability. The antifungal evaluation demonstrated that complexation led to a significant enhancement of biological activity, as shown by the increase in inhibition zone diameters from approximately 14–24 mm for the free forms to 40–50 mm for the β-cyclodextrin complexes. Thus, from ketoconazole, three derivatives were obtained, while in their β-cyclodextrin-complexed forms they exhibited the strongest activity against the tested fungal strains. These findings suggest that the structural modulation of ketoconazole provides an opportunity to develop new compounds with potential applications in antifungal therapy [68].

**Figure 3:**
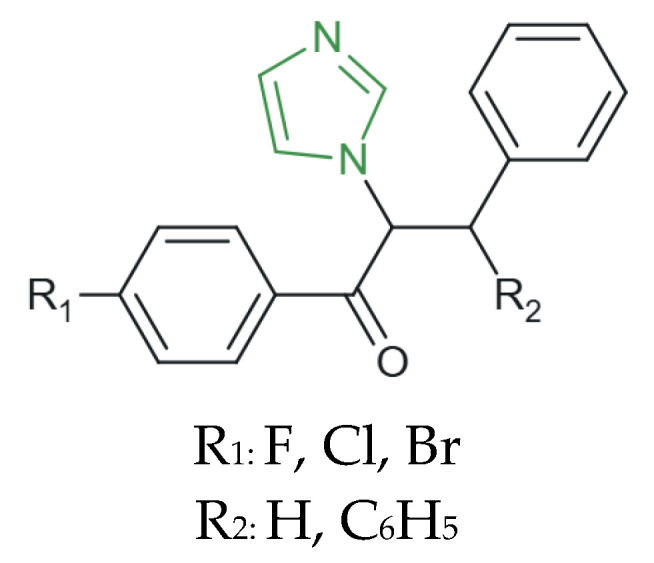
Structural representation of novel antifungal imidazole derivatives [67].

Furthermore, Sadeghian et al. synthesized a novel series of imidazole derivatives (Figure 3) and investigated their in vitro antifungal activity against a panel of clinically relevant fungal strains, including *C. albicans*, *C. tropicalis*, *C. glabrata*, *C. krusei*, *C. dubliniensis*, *C. parapsilosis*, and *C. neoformans*. The evaluated compounds exhibited MIC values in the range of 0.5–16 µg/mL, indicating a broad spectrum of antifungal potency within the series. Notably, derivatives bearing fluorine or bromine substituents at the para-substitution of the benzoyl ring displayed the highest antifungal efficacy, with MIC values as low as 0.5 µg/mL, exceeding the activity of the reference drug fluconazole (1–4 µg/mL) due to the increased lipophilicity imparted by para-halogen substitution, which is likely to facilitate improved penetration into fungal cells and, consequently, a more effective interaction with intracellular targets [67].

**Figure 4:**
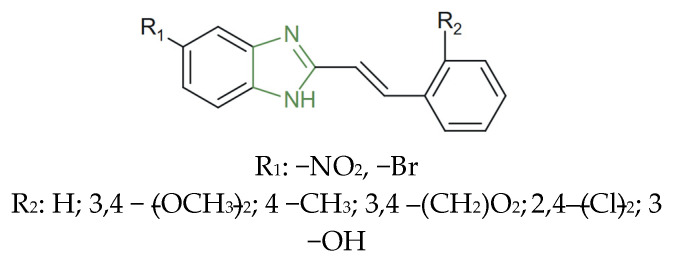
Structural representation of antifungal 5-(nitro/bromo)-styryl-2-benzimidazoles derivatives [69].

On the other hand, Shingalapur et al. synthesized a new series of novel 5-(nitro/bromo)-styryl-2-benzimidazoles (as presented in Figure 4) and evaluated them for in vitro antifungal activity against *C. albicans* and *A. fumigatus* fungal strains, relative to the fluconazole reference compound. A quantitative evaluation indicated that the newly synthesized derivatives exhibited inhibition zones ranging between 18 and 30 mm, which exceeded those of fluconazole (14–20 mm) under identical assay conditions. The brominated analogues displayed the most pronounced activity, showing up to a 40–50% increase in antifungal potency relative to the reference drug, due to the presence of electron-withdrawing nitro and bromo substituents on the styryl moiety [69].

**Figure 5:**
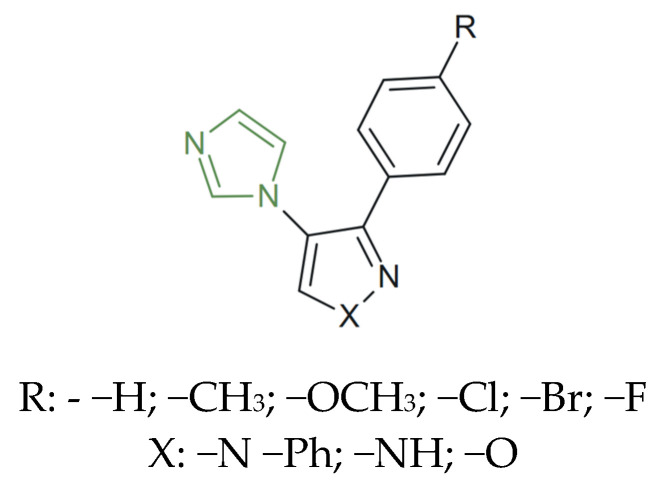
Chemical structure of novel antifungal imidazole–isoxazole/pyrazole hybrids [70].

Remarkable results were also presented by Deshmukh et al. As presented in Figure 5, a series of novel imidazole-clubbed isoxazole/pyrazole derivatives were synthesized and evaluated for their antifungal activities against *C. albicans*, *A. fumigatus*, and *A. niger*. A quantitative evaluation demonstrated inhibition percentages between 54% and 87% across the tested species. Among them, the 4-brominated compound exhibited the highest potency, showing 79% inhibition against *C. albicans*, 87% against *A. fumigatus*, and 87% against *A. niger*, values closely approaching those of the reference antifungal agents fluconazole (94–90%) and clotrimazole (99–96%). The 4-fluoro derivative exhibited excellent antifungal activity against *C. albicans* and a superior potency against *A. niger*, suggesting that imidazole-clubbed hybrids could lead to a series of new compounds, holding promise for the development of new antifungal therapeutic strategies [70].

Overall, SAR trends across antifungal imidazole derivatives highlight the beneficial role of electron-withdrawing substituents and pharmacophore hybridization in enhancing antifungal potency. Furthermore, strategies improving bioavailability, such as β-cyclodextrin complexation, significantly amplify biological activity, underscoring the combined impact of structural and formulation-based optimization.

### 2.2. Antibacterial Activity

The antibacterial activity of imidazole ring-containing compounds is primarily associated with their ability to disrupt the key enzymatic systems involved in bacterial DNA replication [68]. Specifically, many such derivatives interfere with the function of DNA gyrase and topoisomerase IV [71,72,73], enzymes crucial for maintaining DNA topology during cell division. These interactions are thought to involve the imidazole ring establishing favorable binding within the ATP-binding domain of the enzymes, thereby hindering their catalytic action. Additionally, imidazole-based molecules have been shown to stabilize DNA–enzyme cleavage complexes, which promotes accumulation of DNA damage and ultimately leads to bacterial cell death [74]. Beyond targeting DNA replication machinery, several imidazole derivatives have also demonstrated inhibitory activity against other bacterial enzymes, such as β-lactamases and urease [75,76], thereby suggesting a broad-spectrum mechanism [77].

Metronidazole, a well-known imidazole derivative, exerts its antibacterial activity through a mechanism intricately associated with its chemical structure, particularly the nitroimidazole moiety [78,79]. Under anaerobic conditions, the nitro group undergoes reductive activation by microbial intracellular electron transport proteins, leading to the formation of reactive nitro radical intermediates which induce DNA strand cleavage and disrupt nucleic acid synthesis, ultimately resulting in bacterial cell death [80,81].

**Figure 6:**
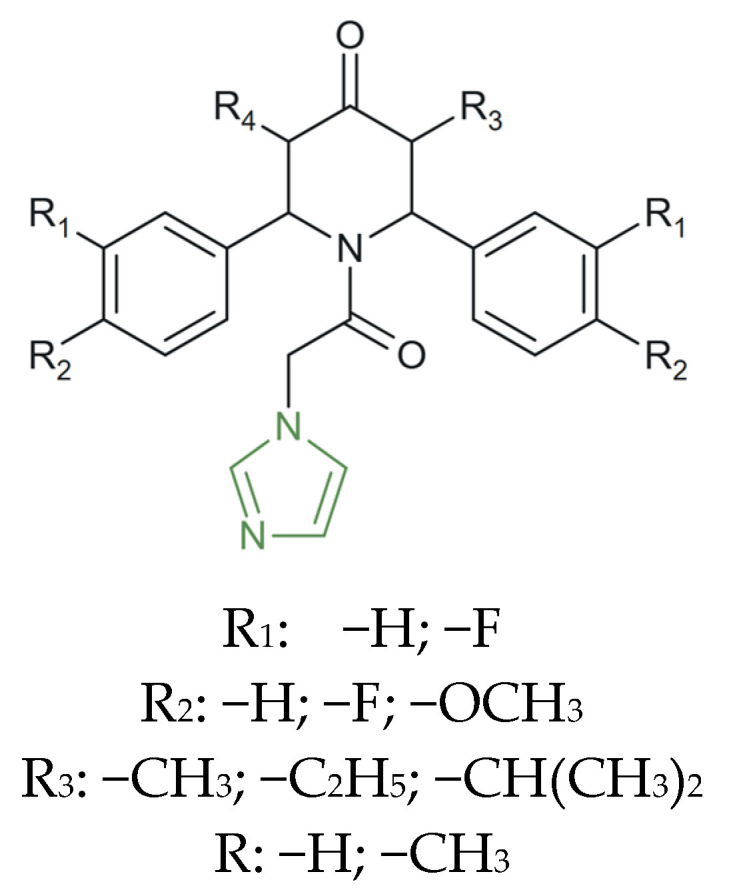
Chemical structure of antibacterial imidazole derivatives [71].

Ramachandran et al. synthesized a series of *N*1-imidazole-acetyl-piperidin-4-one compounds, as presented in Figure 6. The most potent antibacterial effects were observed for derivatives bearing isopropyl and methyl groups at the C-3 and C-5 positions of the piperidinone ring, as well as for those containing para-fluorophenyl substituents at the C-2 and C-6 positions, by exerting the highest level of antibacterial effect against *E. coli*. These structural features afforded minimal inhibitory concentrations ranging between 6.25 and 12.5 µg/mL, indicating strong inhibition against both Gram-positive and Gram-negative bacteria. The isopropyl- and methyl-substituted analogues showed pronounced activity against *E. coli* (MIC = 6.25 µg/mL), whereas the para-fluorophenyl-substituted compound demonstrated superior inhibition of *B. subtilis* (MIC = 6.25 µg/mL), comparable to that of streptomycin (MIC = 12.5 µg/mL) [71].

**Figure 7:**
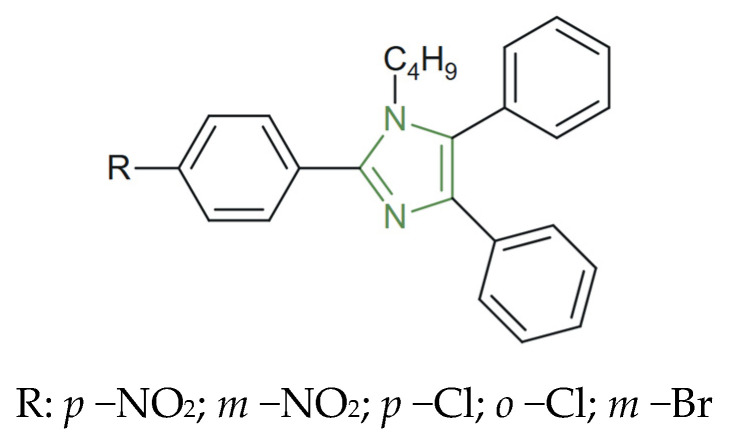
Chemical structure of antibacterial *N*–butyl–imidazole derivatives [72].

On the other hand, Jain et al. synthesized 2-substituted–4,5–diphenyl–*N*–butyl-imidazole derivatives (as presented in Figure 7), obtaining interesting results related to their antibacterial activity against *S. aureus*, *B. subtilis*, and *E. coli*, and compared them to norfloxacin as the positive standard. The biological screening revealed that all tested derivatives displayed moderate activity at 50 µg/mL, with inhibition zones ranging from 3 to 7 mm (14–33%) against *S. aureus* and *B. subtilis*, while increasing the concentration to 150 µg/mL produced zones of 6–9 mm (28–43%). Norfloxacin, used as reference, exhibited a constant inhibition zone of 21 mm at 50 µg/mL, confirming its higher potency, particularly against *E. coli*. Nonetheless, derivatives containing nitro groups at the para-position of the phenyl ring demonstrated a marked improvement in antibacterial efficacy, reaching up to 42–43% inhibition—approximately double that of the unsubstituted analogues. This enhancement is attributed to the electron-withdrawing nature of the –NO_2_ substituent, which increases the overall lipophilicity and facilitates interaction with bacterial membranes [72,73].

The series of 5-(nitro/bromo)-styryl-2-benzimidazoles (previously presented in Figure 4) was evaluated for in vitro antibacterial activity against *S. aureus*, *E. coli*, *E. faecalis*, and *K. pneumoniae* bacterial strains, emphasizing the antibacterial activity of all compounds. The most active analogues, the compounds containing a para-nitro substituent, demonstrated enhanced efficacy, with MIC values of 1 µg/mL against *S. aureus* and 4 µg/mL against *E. coli* and *K. pneumoniae*, comparable to ciprofloxacin (MIC ≈ 0.19–0.78 µg/mL); the bromo-substituted compounds also exhibited balanced activity against both bacterial groups [69].

Therefore, electron-withdrawing groups, particularly nitro, fluoro, and bromo substituents, consistently enhanced antibacterial potency, as evidenced by lower MIC values and increased inhibition zones against both Gram-positive and Gram-negative strains [73,81]. Substitutions that increased lipophilicity, such as isopropyl or methyl groups on aliphatic moieties, further contributed to improved membrane interaction and antibacterial efficacy. Additionally, the presence of nitroimidazole motifs enabled redox-mediated DNA damage under specific conditions, while hybridization strategies and aromatic substitution patterns facilitated effective interaction with key bacterial enzymes, including DNA gyrase and topoisomerase IV.

### 2.3. Anti-Inflammatory and Antiplatelet Activity

**Figure 8:**
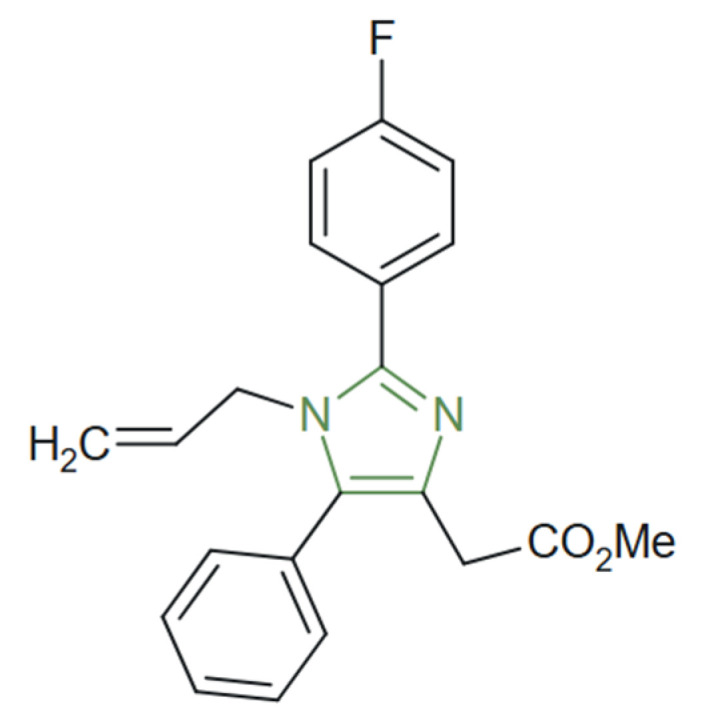
Chemical structure of fluorophenyl imidazole [84].

Imidazole-based compounds exert their anti-inflammatory effects through multiple, well-documented molecular mechanisms. One of the primary modes of action involves the inhibition of cyclooxygenase enzymes, particularly COX-2, resulting in the suppression of prostaglandin synthesis and, consequently, the attenuation of pain and inflammation [47,48]. In addition to COX inhibition, several imidazole derivatives have also demonstrated dual inhibition of COX-2 and 5-lipoxygenase (5-LOX), effectively reducing both prostaglandin and leukotriene levels [82]. Beyond the enzymatic inhibition effect, certain derivatives have been shown to suppress the activation of neutrophils, to reduce reactive oxygen species (ROS) production [83], and to inhibit the release of inflammatory mediators such as myeloperoxidase (MPO) and interleukin-6 (IL-6). At the transcriptional level, imidazole scaffolds (Figure 8) can inhibit the NF-κB signaling pathway by blocking the nuclear translocation of the p65 subunit, leading to a decreased expression of pro-inflammatory cytokines, including TNF-α, IL-1β, and IL-6 [84,85]. Furthermore, some imidazole analogues target the p38 mitogen-activated protein kinase (MAPK) pathway [82], interfering with intracellular inflammatory signaling cascades, while in certain cases their anti-inflammatory action is enhanced by the ability of the imidazole ring to chelate metal ions essential for the catalytic activity of metalloenzymes involved in inflammation [86,87]. These diverse mechanisms collectively underscore the therapeutic potential of structurally optimized imidazole derivatives in modulating acute and chronic inflammatory processes.

**Figure 9:**
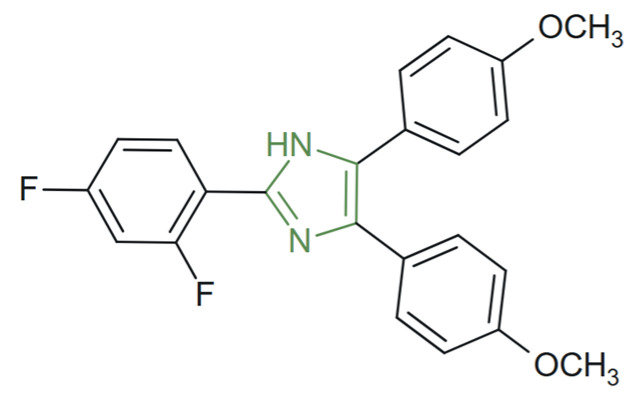
Chemical structure of fenflumizole [82].

Likewise, the research conducted by Aono et al. compared the antiplatelet effect of fenflumizole (Figure 9) with aspirin or ticlopidine. The compound exhibited half-maximal inhibitory concentrations of 4.0 × 10^−7^ M for arachidonate and 1.7 × 10^−7^ M for collagen-induced aggregation, compared with 1.4 × 10^−4^ M and 5.3 × 10^−5^ M for aspirin and ticlopidine. In in vivo assays on rabbits, oral administration of fenflumizole (0.3–3 mg/kg) resulted in a marked, dose-dependent suppression of platelet aggregation up to 24 h post-treatment, whereas ticlopidine required doses of 100–300 mg/kg to achieve comparable effects [82].

**Figure 10:**
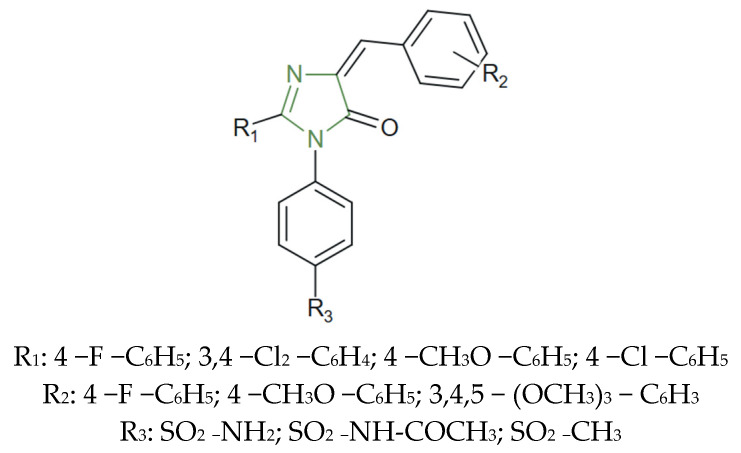
Chemical structure of novel COX-2 inhibitory imidazole derivatives [45].

A comparable effect was also observed by Rayan et al., who demonstrated significant COX-2 inhibitory activities by imidazole and derivatives bearing various substituents, as presented in Figure 10. Structural modifications, such as the introduction of sulfonyl, methoxy, or other various moieties, have been shown to enhance COX-2 selectivity, potency, and gastric tolerability. Among these, derivatives structurally related to celecoxib exhibited the most promising biological profiles, with several compounds displaying IC_50_ values comparable to the standard drug, indomethacin [45].

**Figure 11:**
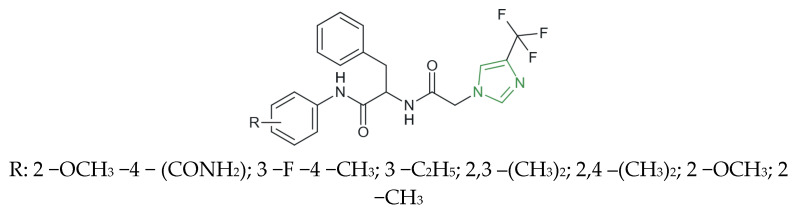
Chemical structure of novel *N*-substituted [4-(trifluoro methyl)-1*H*-imidazole-1-yl] amide derivatives [86].

Awasthi et al. synthesized a novel series of *N*-substituted [4-(trifluoromethyl)-1*H*-imidazol-1-yl] amide derivatives (Figure 11) and evaluated their in vitro anti-inflammatory activity through inhibition of p38 mitogen-activated protein kinase. The synthesized compounds exhibited IC_50_ values ranging from 1246.56 to 403.57 nM, indicating variable inhibitory potency across the series. Among them, the 2,4-dimethoxy-substituted derivative emerged as the most active compound, displaying considerable p38 kinase inhibitory activity with an IC_50_ value of 403.57 nM, which is comparable to that of the reference prototype inhibitor adezmapimod (SB203580) (IC_50_ = 222.44 nM). These experimental findings were further supported by in silico molecular docking studies, in which, from the series of derivatives, the same compound achieved the most favorable binding score (−7.83 kcal/mol) within the p38 MAP kinase active site, corroborating its enhanced inhibitory profile [86].

Overall, imidazole derivatives bearing para-substituted aromatic rings, particularly fluorophenyl and methoxyphenyl moieties, consistently exhibited enhanced anti-inflammatory activity compared to unsubstituted analogues.

The introduction of sulfonyl-containing groups, such as –SO_2_NH_2_, –SO_2_NHCOCH_3_, or –SO_2_CH_3_, was associated with increased potency and selectivity, suggesting that such functionalities represent favorable pharmacophoric elements within anti-inflammatory imidazole scaffolds. Additionally, electron-withdrawing substituents, including fluoro and chloro groups, generally improved biological activity, whereas voluminous substitutions without electronic contribution showed no consistent benefit [45].

The key advantage of these imidazole derivatives lies in their selective modulation of inflammatory signaling, particularly the NF-κB pathway, rather than the indiscriminate suppression of essential immune functions. By targeting upstream inflammatory signals and preferentially acting on activated immune cells, these compounds exhibit a more selective anti-inflammatory profile with reduced potential for systemic adverse effects. Importantly, the findings reported by Rocha et al. highlighted the selective anti-inflammatory action of imidazole alkaloids, which preferentially target activated neutrophils and NF-κB-mediated signaling without inducing generalized immunosuppression [84].

Similarly, the studies published by dos Santos Nascimento et al. [85] and Awasthi et el. [86] demonstrated that newly synthesized imidazole derivatives exert anti-inflammatory effects through modulation of the p38 MAPK signaling pathway, leading to the suppression of pro-inflammatory mediator production and confirming the role of signaling pathway interference in the anti-inflammatory activity of imidazole-based compounds.

### 2.4. Antitubercular Activity

Imidazole-based antitubercular agents exert their effects via a prodrug activation mechanism that generates highly reactive intermediates capable of disrupting key pathways in *M. tuberculosis*. Bicyclic nitroimidazoles such as delamanid and pretomanid are reductively activated by the mycobacterial deazaflavin-dependent nitroreductase, which acts by inhibiting the synthesis of mycobacterial cell wall components, methoxy mycolic acid, and ketomycolic acid, through the F420 coenzyme mycobacteria system and by generating nitrous oxide [48,49,50]. Recent findings have further elucidated that the activated metabolites of these agents inhibit the essential enzyme DprE2, a key reductase involved in arabinogalactan biosynthesis for the mycobacterial cell wall [88,89,90,91].

Additionally, a comprehensive review by Fan et al. underscored the versatility of imidazole-containing derivatives, including both nitroimidazoles and related hybrid scaffolds, in exhibiting potent in vitro and in vivo activity against drug-sensitive and drug-resistant *M. tuberculosis* strains [89]. Structural innovations such as 1*H*-benzo[d]imidazole derivatives have also demonstrated the capacity to interfere with mycolic acid metabolism, adding to the mechanistic diversity of imidazole-based antimycobacterials [91]. Imidazole-containing derivatives possess a wide range of biological properties, among which several have demonstrated notable antitubercular activity, a prominent example being 4-nitroimidazole delamanid or pretomanid, which has received clinical approval for the treatment of patients infected with multidrug-resistant *M. tuberculosis* [89,90].

**Figure 12:**
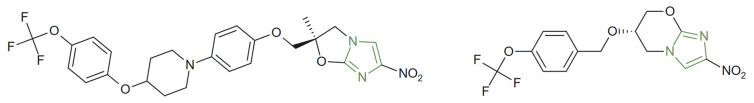
Chemical structures of delamanid and pretomanid [90].

In vitro and in vivo studies have showed that delamanid (Figure 12) has a pronounced antibacterial activity against drug-sensitive and drug-resistant strains of *M. tuberculosis*.

The study conducted by Shingalapur et al. also reported the synthesis of a series of 5-(nitro/bromo)-styryl-2-benzimidazoles (shown above in Figure 4) and the in vitro evaluation against *M. tuberculosis* H37Rv. A quantitative assessment showed that the bromo-substituted analogues exhibited distinctly superior activity by inhibiting *M. tuberculosis* growth by 83%, 63%, 76%, and 54% for the analogues bearing unsubstituted, 4-CH_3_, 2,4-Cl, and 3,4-OCH_3_ phenyl moieties, respectively, at a test concentration of 7.25 µg/mL. By contrast, the nitro-containing derivatives displayed inhibition values below 50%, therefore showing only moderate or negligible inhibitory potential [69].

### 2.5. Anti-Ulcer Activity

Imidazole derivatives have garnered significant attention as potential anti-ulcer agents, owing to their multifactorial mechanisms of action, which extend beyond mere acid suppression to include mucosal protection, anti-*H. pylori* activity, and modulation of oxidative stress pathways [92,93,94]. A prominent example is omeprazole, a benzimidazole-based proton pump inhibitor (PPI) which undergoes acid-catalyzed conversion in the parietal cell canaliculi to a sulfenamide intermediate that covalently binds to cysteine residues on the H^+^/K^+^-ATPase enzyme, thereby irreversibly inhibiting gastric acid secretion [95,96,97,98,99,100]. However, newer imidazole derivatives extend the therapeutic paradigm by exhibiting urease-inhibitory activity, thus targeting *H. pylori* more directly.

**Figure 13:**
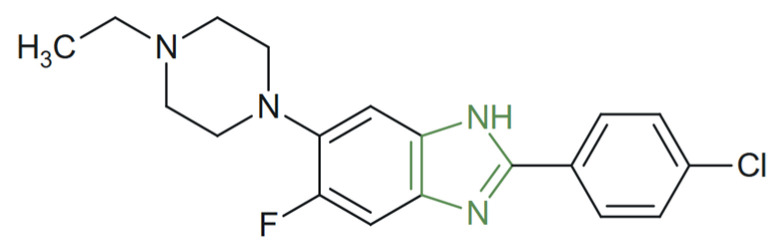
Chemical structure of new anti-ulcer benzimidazole derivative [96].

For instance, Moghadam et al. synthesized a new benzimidazole derivative, as presented in Figure 13, that demonstrated potent urease inhibition and substantial in vitro activity against *H. pylori*, with IC_50_ values ranging from 3.36 to 10.81 µM—significantly stronger than those of the reference inhibitors thiourea (IC_50_ = 22 µM) and hydroxyurea (IC_50_ = 100 µM), indicating dual mechanisms involving both bacterial eradication and acid reduction [96].

**Figure 14:**
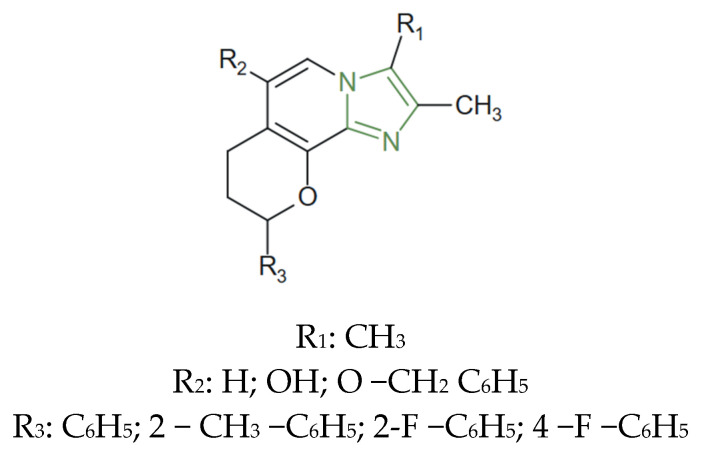
Chemical structure of anti-ulcer imidazo[1,2-a]pyridine [99].

Moreover, some imidazole-based agents have been shown to attenuate oxidative damage to gastric mucosa, either by scavenging reactive oxygen species or by enhancing endogenous antioxidant enzyme systems, such as glutathione peroxidase and superoxide dismutase, thereby reducing lipid peroxidation and preserving mucosal integrity. Additional studies have identified imidazo[1,2-a]pyridine (Figure 14) derivatives capable of inhibiting gastric lesions induced by NSAIDs or by stress via both antisecretory and cytoprotective mechanisms [99].

These findings highlight the therapeutic versatility of imidazole sulfones, which act through a multifaceted anti-ulcer mechanism involving acid suppression [100,101], bacterial eradication [100], urease inhibition [96,99,100,101,102], and antioxidant-mediated mucosal protection, thereby positioning this structural class as a promising platform for the development of next-generation anti-ulcer therapeutics.

**Figure 15:**
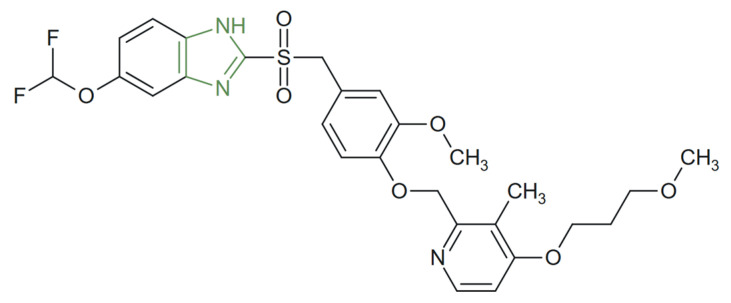
Chemical structure of anti-ulcer benzimidazole sulfone derivatives [54].

Recent investigations into benzimidazole sulfones (Figure 15) conducted by Rajesh et al. have further underscored the therapeutic relevance of imidazole derivatives in the management of acid-related disorders through targeted inhibition of the gastric H^+^/K^+^-ATPase enzyme. Particularly, this imidazole derivative demonstrated superior potency, achieving 94.8% inhibition, surpassing the reference drug omeprazole, which showed 80.0% relative inhibition [54].

### 2.6. Antihyperglycemic Activity

Imidazole derivatives can modulate multiple molecular targets involved in glucose metabolism, such as the inhibition of key enzymes, for example α-glucosidase, thereby reducing postprandial hyperglycemia through delayed carbohydrate digestion and glucose absorption in the small intestine [61,103,104]. Moreover, several imidazole compounds have demonstrated potent inhibitory activity against protein tyrosine phosphatase 1B, a negative regulator of insulin signaling, thus enhancing insulin sensitivity by sustaining the phosphorylation of the insulin receptor and its downstream signaling components. In addition, some imidazole derivatives have been reported to activate AMP-activated protein kinase, a master regulator of cellular energy homeostasis, which facilitates increased glucose uptake and fatty acid oxidation in peripheral tissues. A few studies have further suggested that certain structural analogues may also interfere with dipeptidyl peptidase 4 activity [104,105,106], prolonging incretin hormone action and enhancing glucose-stimulated insulin secretion. Collectively, these findings underscore the multifunctional nature of imidazole derivatives in addressing key pathophysiological features of type 2 diabetes and support their continued development as scaffolds for next-generation antihyperglycemic therapeutics.

**Figure 16:**
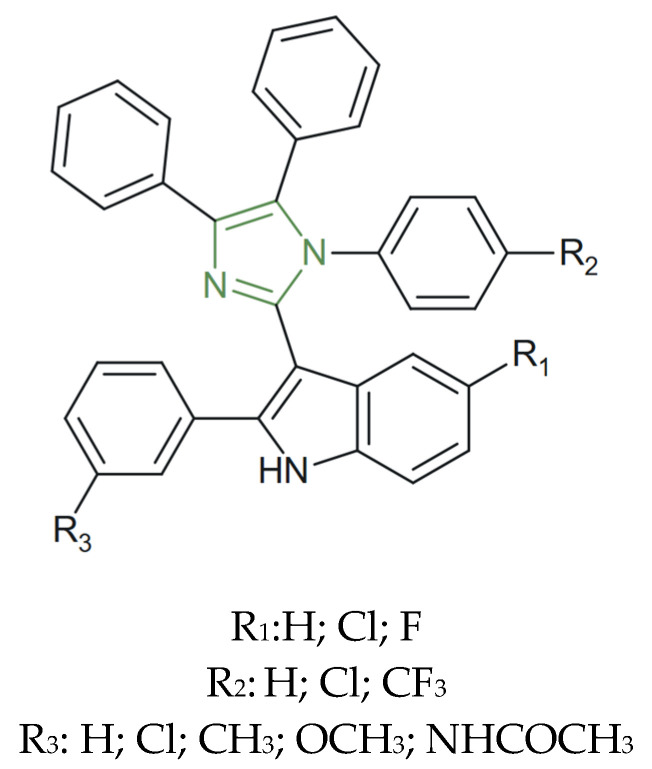
Chemical structure of antihyperglycemic triarylimidazoles [105].

In this regard, Naureen et al. reported the design and synthesis of a series of triarylimidazoles tethered with 2-arylindoles via a one-pot multicomponent condensation approach, as presented in Figure 16. The compounds were evaluated for α-glucosidase inhibition and, among them, the derivative incorporating fluorine and an acetamide substituent exhibited the strongest activity, showing 98.71 ± 0.15% inhibition and an IC_50_ of 8.37 ± 0.11 µM—significantly stronger than the reference drug acarbose (IC_50_ = 37.25 ± 0.15 µM), suggesting a synergistic role of these groups in enhancing enzyme inhibition [105].

**Figure 17:**
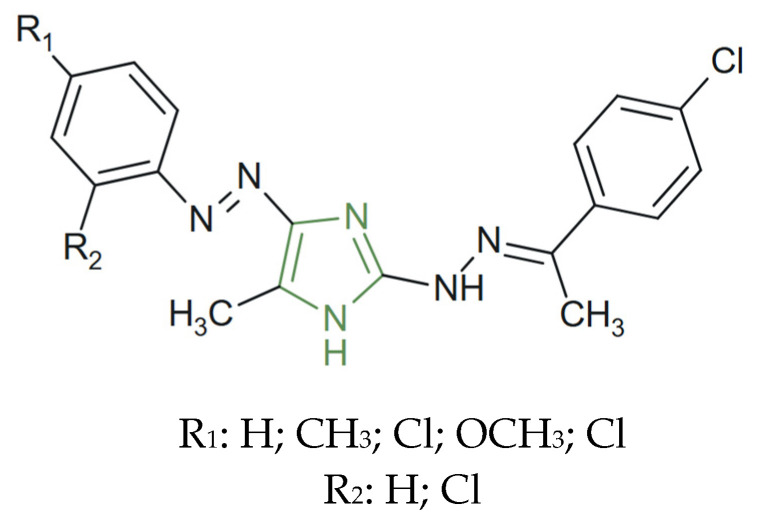
Chemical structure of new antihyperglycemic hydrazone–imidazole derivatives [61].

On the other hand, to obtain the compounds presented in Figure 17, a series of novel hydrazone–imidazole derivatives were synthesized by Badrey et al. as potential α-amylase inhibitors for type 2 diabetes management. The findings of this in silico study support the potential of imidazole-based scaffolds as antihyperglycemic agents. Among the synthesized analogues, compounds bearing electron-withdrawing groups, such as chloro- and nitro-substituents, exhibited the highest α-amylase inhibitory potency, with predicted binding energies ranging from −9.2 to −11.4 kcal mol^−1^ in molecular docking studies—values that surpassed those of the standard drug acarbose (−8.7 kcal mol^−1^) [61].

Therefore, these antihyperglycemic imidazole derivatives demonstrate that the nature of aromatic substitution and the presence of electron-withdrawing functionalities are key determinants of biological potency. In triarylimidazole systems tethered with indole moieties, fluorine substitution and acetamide-containing groups were associated with markedly enhanced α-glucosidase inhibitory activity, yielding IC_50_ values significantly lower than those of the reference drug acarbose [105].

Similarly, in hydrazone–imidazole derivatives evaluated as α-amylase inhibitors, electron-withdrawing substituents, such as chloro and nitro groups, consistently correlated with superior predicted inhibitory potency compared with unsubstituted or electron-donating analogues [61].

### 2.7. Antidepressant Activity

Imidazole-based scaffolds have gained recognition as privileged structural motifs in the development of novel antidepressant agents, primarily due to their capacity to modulate monoaminergic neurotransmission through diverse molecular mechanisms. The electron-rich nature of the imidazole ring, combined with its ability to engage in π–π stacking, hydrogen bonding, and metal ion coordination, imparts strong binding affinity toward the critical protein targets implicated in serotonergic and noradrenergic pathways [107,108,109]. In this context, mechanistic studies have suggested that imidazole-based derivatives can inhibit serotonin transporter and, in some cases, the norepinephrine transporter (NET), thereby increasing the synaptic availability of monoamines, a hallmark mechanism underlying classical antidepressant therapies. In addition, specific substitution patterns on the imidazole nucleus have been reported to enhance selectivity for 5-HT1A_{1A}1A and 5-HT2A_{2A}2A receptor subtypes, indicating a dual mode of action involving both transporter blockade and direct receptor modulation. Molecular docking analyses and SAR evaluations have further demonstrated that the imidazole moiety facilitates optimal orientation of the ligand within the binding pockets of serotonergic targets, ensuring the stable π–π stacking, halogen bonding, and hydrogen interactions essential for antidepressant efficacy. Electron-withdrawing substituents, such as fluoro-, chloro-, or trifluoromethyl groups, enhance receptor affinity by strengthening halogen–π interactions with residues like Ser5.42., whereas small alkyl substituents like methyl or ethyl groups at the N-1 or C-3 positions contribute to an increased activity by improving π–π interactions with Tyr2.64 amino acid residue. Additionally, flexible piperazinyl or alkyl linkers orient the imidazole nucleus for dual interaction with 5-HT_1_A/5-HT_7_ receptors and serotonin transporters SERT, thus enhancing the multi-target pharmacological profile [30,110,111,112,113,114,115,116].

**Figure 18:**
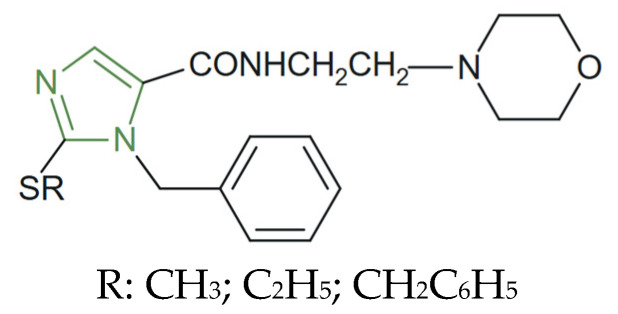
Chemical structure of moclobemide analogues [114].

Starting from the idea of developing imidazole derivatives with an antidepressant effect, Hadizadeh et al. synthesized some moclobemide analogues, as shown in Figure 18, by replacing the moclobemide phenyl ring with substituted imidazole. The authors studied their antidepressant activity using a forced swimming test, while some of the synthesized analogues, such as the methyl- and ethyl-substituted thioimidazole analogues, showed significant reductions in immobility time even at low doses (2.5–10 mg/kg i.p.), while the benzyl-substituted analogue maintained strong activity across all tested doses (2.5–20 mg/kg i.p.), outperforming moclobemide, which is only effective at higher doses (≥30 mg/kg i.p.) [114].

**Figure 19:**
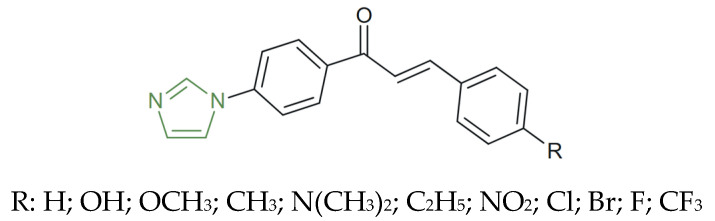
Chemical structure of novel imidazole–chalcones [115].

Moreover, the imidazole-based chalcones obtained by Sasidharan et al. exhibited preferential inhibition of MAO-B over MAO-A, with para-substituents on the aromatic ring, as presented in Figure 19, exerting a decisive influence on potency and selectivity. Among them, the derivate bearing a dimethylamine group showed strong MAO-A inhibition, with IC_50_ values of 0.30 ± 0.01 µM (MAO-A) and 0.40 ± 0.02 µM (MAO-B), while the brominated derivative showed IC_50_ = 0.42 ± 0.01 µM and a selectivity index of 9.6, demonstrating a marked preference for MAO-B inhibition and therefore emerging as the most selective MAO-B inhibitor, suggesting that these derivatives have potential as dual modulators of monoaminergic pathways relevant to antidepressant therapy [115]. Also, compounds featuring hydroxyl, chlorine, or trifluoromethyl substituents on the imidazole–chalcone framework exhibited significantly reduced inhibitory activity toward both isoforms, indicating that strong electron-withdrawing groups decrease affinity for the enzyme active site [113,114,115].

This analysis of imidazole-based antidepressant agents indicates that substitution at the imidazole core and the nature of peripheral aromatic substituents are critical determinants of potency and selectivity in moclobemide-inspired imidazole analogues; small alkyl substituents, such as methyl and ethyl groups, as well as benzyl substitution, were associated with markedly enhanced antidepressant activity, allowing significant reductions in immobility time at lower doses compared with the parent drug, also suggesting that moderate lipophilicity and flexible substituents favor improved pharmacological performance in this scaffold [114].

In imidazole–chalcone derivatives, SAR trends revealed a strong dependence of monoamine oxidase inhibition on the para-substitution of the aromatic ring, such as dimethylamine groups, which exhibited high inhibitory potency toward MAO enzymes, whereas halogen substitution, especially bromine, conferred enhanced selectivity for MAO-B [115]. By contrast, derivatives containing strong electron-withdrawing groups, such as hydroxymethyl, chloromethyl, or trifluoromethyl substituents, consistently showed reduced inhibitory activity, indicating that excessive electron withdrawal is unfavorable for antidepressant potency in this framework.

### 2.8. Antiparasitic Activity

Imidazole-derived scaffolds have earned a long-standing reputation in antiparasitic chemotherapy, displaying effective activity against both protozoan and helminth infections. Among the earliest and most clinically transformative examples are the 5-nitroimidazoles—notably metronidazole, tinidazole, and ornidazole—which revolutionized the treatment of protozoal diseases, such as those caused by *E. histolytica*, *G. lamblia*, and *T. vaginalis* [117,118,119,120]. Metronidazole remains the prototypical nitroimidazole, characterized by a 5-nitroimidazole core that confers a balance of lipophilicity for membrane diffusion and hydrophilicity for systemic bioavailability, while functioning as a prodrug that undergoes reductive bioactivation within the anaerobic or microaerophilic environment of susceptible protozoa. The nitro group of metronidazole serves as an electron acceptor for low redox-potential enzymes, such as ferredoxin-dependent nitroreductases, generating highly reactive nitro radical anions which interact with nucleophilic cellular targets, forming covalent adducts with DNA bases and thiol-containing proteins, ultimately leading to strand cleavage, loss of helical integrity, and inhibition of nucleic acid synthesis [120,121,122,123]. Additionally, the oxidative stress induced by reactive nitrogen species contributes to mitochondrial dysfunction and depletion of cellular thiols, exacerbating cytotoxicity in anaerobes while sparing aerobic host cells, thereby explaining the selective toxicity of metronidazole arising from its redox-dependent mechanism, which is only activated under the low oxygen tensions characteristic of parasitic and bacterial pathogens. This redox-conditional pharmacology has inspired numerous structural analogues and hybrid scaffolds seeking to enhance potency, overcome resistance, and broaden the antiparasitic spectrum of nitroimidazole-based chemotypes.

**Figure 20:**
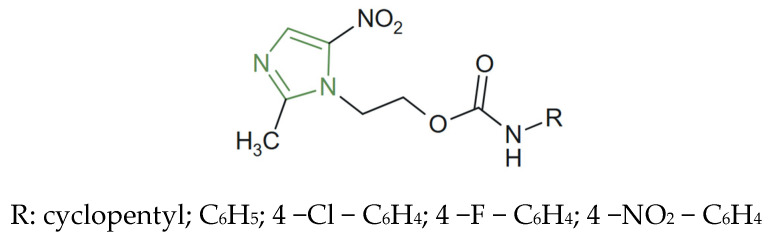
Chemical structure of novel antiparasitic imidazole derivatives [123].

In this regard, Martínez-Rosas et al. developed a series of imidazole carbamate derivatives (Figure 20) as alternatives to nitroimidazoles for *T. vaginalis*. Among the novel derivatives, the compound bearing an *N*-phenylcarbamate substituent linked to a methyl-substituted imidazole ring displayed the most potent trichomonacidal activity, with an IC_50_ of 4.1 µM, surpassing the reference drug metronidazole (IC_50_ = 6.8 µM) [123].

**Figure 21:**
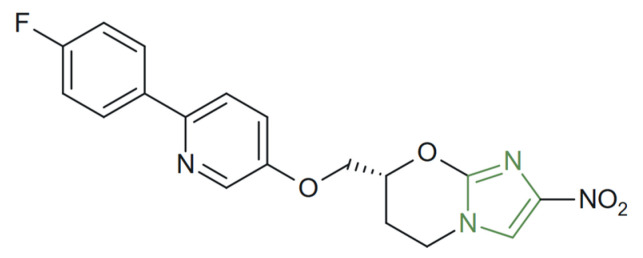
Chemical structure of novel antiparasitic imidazole derivatives [121].

In another study, Van Bocxlaer et al. investigated a panel of newly synthesized nitroimidazole derivatives (Figure 21) and identified a promising candidate for the treatment of *Leishmania* infections, which exhibited potent inhibitory activity with an EC_50_ value of 4.56 µM against intracellular amastigotes of *Leishmania* spp., while showing negligible cytotoxicity toward mammalian host cells. An in vivo evaluation in murine models further demonstrated significant reduction of parasite load, confirming the compound’s therapeutic potential [120,121].

By contrast, benzimidazole derivatives, such as albendazole, mebendazole, thiabendazole, and triclabendazole, are widely used as broad-spectrum anthelmintics, binding selectively to the β-tubulin of parasitic helminths and inhibiting polymerization into microtubules [122,123]. By impairing microtubule formation, they disrupt mitosis, cell motility, intracellular transport, glucose uptake, and energy metabolism, leading to the depletion of glycogen stores, the inhibition of larval growth and egg production, the loss of viability, and the eventual death of the parasite [124,125]. Depending on the pattern of substitution—nitro group for protozoan-targeted radical generation, or benzimidazole moiety for tubulin binding—the same heterocyclic nucleus can be repurposed to exploit the fundamentally distinct biochemical vulnerabilities of parasites [126,127,128,129,130].

**Figure 22:**
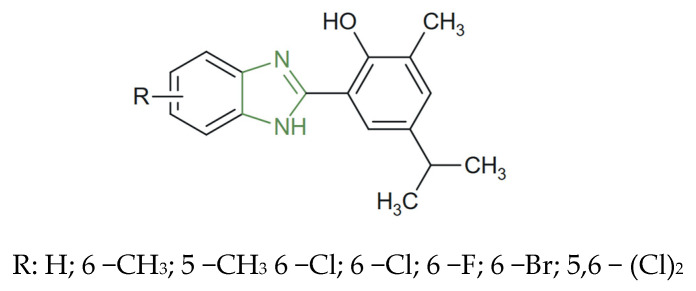
Chemical structure of novel antimalarial 2-aryl-benzimidazole derivatives [127].

In this regard, Bhoi et al. reported the synthesis of a new series of benzimidazole (Figure 22) derivatives obtained through the rearrangement of 2-formyl carvacrol with 1,2-phenylenediamines, affording compounds bearing at the second position of a 2-phenolic moiety substituted with electron-donating alkyl groups. The newly synthesized analogues exhibited remarkable antiparasitic activity, particularly against *P. falciparum*, with IC_50_ values ranging from 1.52 to 3.31 µM. The 5,6-disubstituted derivatives achieved potency comparable to that of the reference antimalarial drug quinidine (IC_50_ = 0.83 µM) [127].

**Figure 23:**
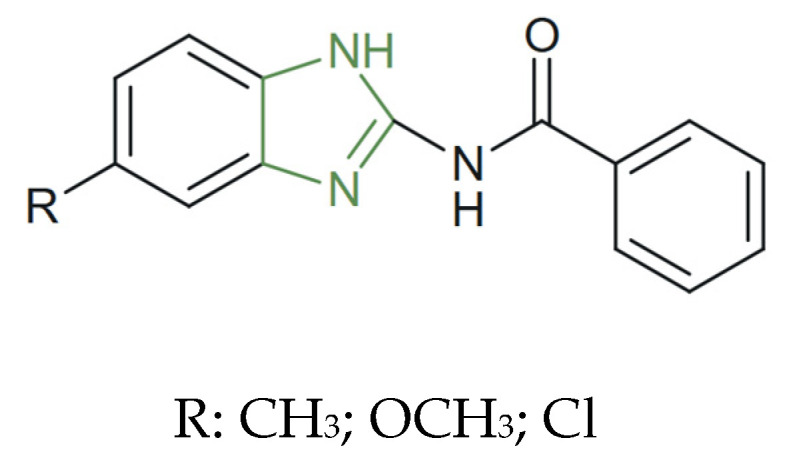
Chemical structure of novel antiparasitic 2-acylamino-benzimidazole derivatives [129].

In a recent study of benzimidazole as an antiparasitic agent, Valderas-García et al. highlighted the importance of benzimidazoles in overcoming drug resistance among trematodes by an in vitro evaluation against multiple developmental stages of *F. hepatica*. Among the tested derivatives (Figure 23), the chloro-substituted benzimidazole derivative exhibited marked ovicidal activity, with >80% inhibition of egg hatching at 10 µM, and sustained efficacy (>70%) even at 5 µM, and substantial adulticidal activity with approximately 60% inhibition of worm motility after 72 h at 10 µM—a significantly stronger effect than that observed for albendazole metabolites under identical conditions [129].

The collected studies indicate that the antiparasitic activity of imidazole and benzimidazole derivatives is strongly influenced by the nature of the heterocyclic core and the pattern of substitution. For protozoan infections, compounds retaining a nitroimidazole framework consistently displayed high efficacy, while alternative imidazole derivatives bearing appropriately substituted carbamate or aromatic moieties were able to match or even surpass the activity of classical nitroimidazoles [130]. In the case of benzimidazole-based antiparasitic agents, enhanced activity was frequently associated with halogenated and 5,6-disubstituted structures, particularly against *P. falciparum* [127].

### 2.9. Anticancer Activity

The imidazole core was identified as essential isostere for pyrazole, triazole, tetrazole, thiazole, and oxazole, for the design of diverse biologically active molecules, having the ability to overcome the different disadvantages of current anticancer agents [131,132,133]. Among imidazole-based derivatives, nitroimidazoles represent a distinct subclass characterized by their high electron affinity and bioreductive activation under hypoxic conditions. These compounds passively diffuse into cells where they are reduced by nitroreductase enzymes to generate reactive intermediates capable of forming covalent adducts with DNA and other cellular macromolecules. Due to their preferential accumulation in O_2_ starved tissue, nitroimidazoles have been pursued as potential hypoxia-directed therapeutics, while 2-nitroimidazoles (the well-known misonidazole or pimonidazole) are used in hypoxic tumor cells because of the correlation with the malignant progression in advanced cervical cancer [62,134,135]. Notably, dacarbazine, a triazene-containing imidazole derivative, acts as a DNA-alkylating agent following metabolic activation to MTIC (5-(3-methyl-1-triazeno)imidazole-4-carboxamide), inducing the cytotoxic methylation of guanine residues and subsequent strand breaks. Similarly, temozolomide, a second generation analog of dacarbazine, features an imidazotetrazine ring system that undergoes spontaneous hydrolytic activation under physiological pH to yield MTIC, achieving improved oral bioavailability and better tumor penetration [136,137], while bendamustine, another clinically validated imidazole derivative, combines a 2-chloroethylamine alkylating warhead with an imidazole nucleus that enhances pharmacokinetic stability and imparts a distinct cytotoxic profile compared to classical nitrogen mustards [138,139].

**Figure 24:**
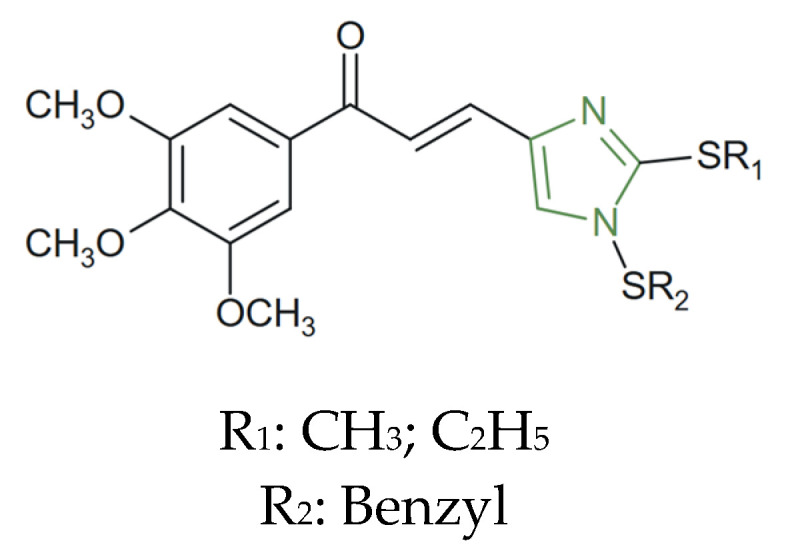
Chemical structure of novel anticancer imidazole–chalcone derivatives [140].

Regarding this research path, Rahimzadeh et al. designed and synthesized novel imidazole–chalcone derivatives (Figure 24) as tubulin polymerization inhibitors by arresting mitosis and inhibiting cancer cell proliferation. The antiproliferative activity of the imidazole–chalcone was assessed on some human cancer cell lines including A549 (adenocarcinoma human alveolar basal). The cytotoxic potential of the synthesized compound was evaluated against several human cancer cell lines, including HeLa (cervical carcinoma), MCF-7 (breast cancer), MCF-7/MX (mitoxantrone-resistant breast cancer), and HepG2 (hepatocellular carcinoma), using 5-fluorouracil as a reference standard. Among the evaluated derivatives, those incorporating a trimethoxy-substituted chalcone ring (3,4,5-OCH_3_) and bearing either a methylthio- or ethylthio- group on the imidazole nucleus displayed enhanced cytotoxicity, particularly against MCF-7 (IC_50_ = 9.88 ± 1.56 µM) and MCF-7/MX (IC_50_ = 20.2 ± 2.76 µM) cells, demonstrating higher cytotoxic activity toward A549 lung cancer cells compared to the other tested cell lines [140].

**Figure 25:**
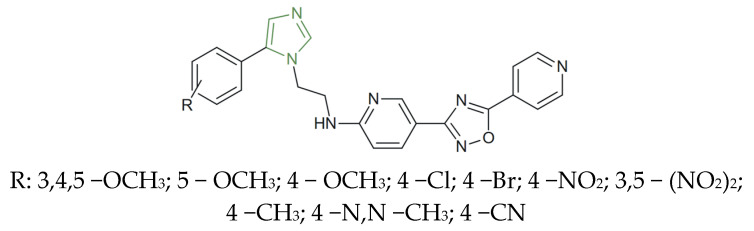
Chemical structure of anticancer imidazole-based pyridine and 1,2,4-oxadiazole derivatives [56].

Vasamsetti et al. synthesized a series of imidazole-based pyridine-1,2,4-oxadiazole derivatives (Figure 25) and demonstrated their significant in vitro anticancer activity. Among the tested compounds, the electron-donating trimethoxy and methoxy derivatives displayed the strongest cytotoxic activity, with IC_50_ values as low as 0.02 ± 0.0047 µM on A549, 0.07 ± 0.0026 µM on MCF-7, and 0.17 ± 0.085 µM on PC-3 cell line, markedly superior to etoposide, which was used as a reference (IC_50_ ≈ 2–3 µM) [56]. Conversely, the introduction of strong electron-withdrawing groups, such as 4-Cl, 4-Br, or nitro moieties, reduced the antiproliferative potency, yielding IC_50_ values above 3–10 µM across the PC-3, A549, and MCF-7 cell lines.

The most active analogues also exhibited nanomolar inhibitory activity against topoisomerase IIβ, EGFR, and VEGFR (IC_50_ = 1.58 ± 0.46 µM, 0.92 ± 0.12 µM, and 1.24 ± 0.36 µM, respectively), further strengthening their antitumor potential through simultaneous interference with multiple oncogenic signaling pathways, indicating the imidazole moiety’s critical role in enhancing cytotoxic efficacy [56].

**Figure 26:**
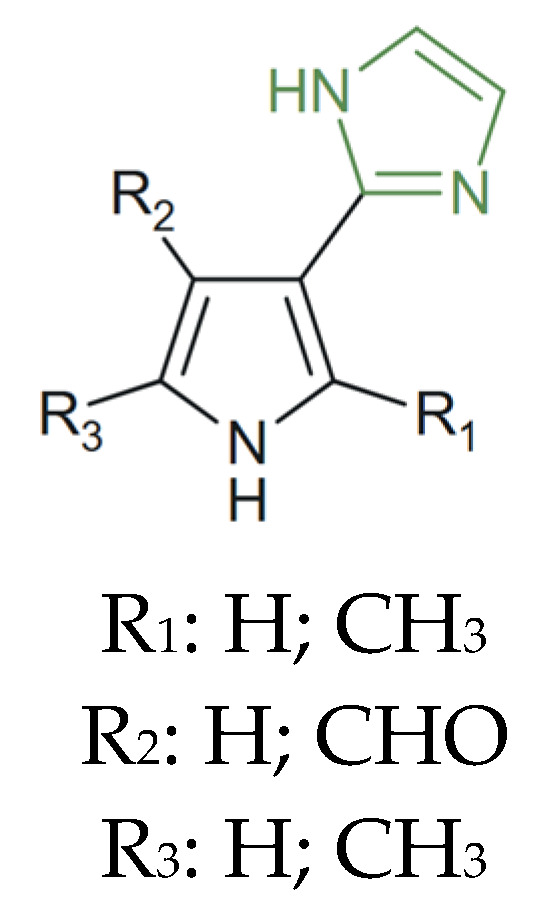
Chemical structure of novel anticancer pyrrolyl–imidazole derivatives [141].

A series of pyrrole–imidazole derivatives (Figure 26) were synthesized and investigated by Ram et al. for their anticancer properties. Molecular docking studies against key targets, including SYK, PI3K, and BTK, demonstrated strong binding affinities across the series, while QSAR analyses highlighted HOMO, LUMO, softness, and chemical potential as critical molecular descriptors associated with antileukemia activity [141].

**Figure 27:**
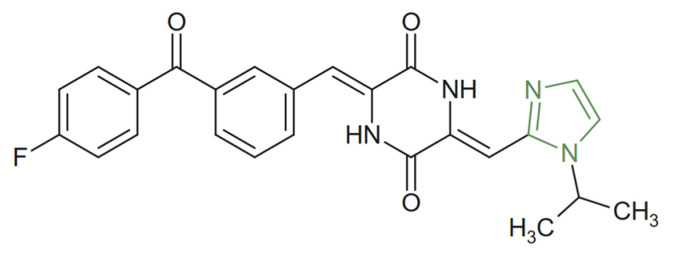
Chemical structure of potent anticancer phenylahistin derivative [59].

In another study, Chen et al. synthesized a series of phenylahistin derivatives incorporating the 1,3-imidazole nucleus to explore their potential as tubulin polymerization inhibitors. SAR studies revealed that substitution at the first position of the imidazole ring with non-chained alkyl groups containing two–three carbon atoms significantly enhanced cytotoxic activity, while longer substituents reduced efficacy. Among these, a compound featuring a 1-isopropyl-1,3-imidazol-2-yl moiety and a para-fluorophenyl or a para-fluorophenoxy (Figure 27) demonstrated potent cytotoxic effects against multiple human cancer cell lines, with IC_50_ values in the nanomolar range—2.40 nM on NCI-H460, 1.61 nM on BxPC-3, and 2.07 nM on HT-29—by inhibiting tubulin polymerization and by inducing p53-dependent apoptosis, as confirmed by immunofluorescence assays [59].

**Figure 28:**
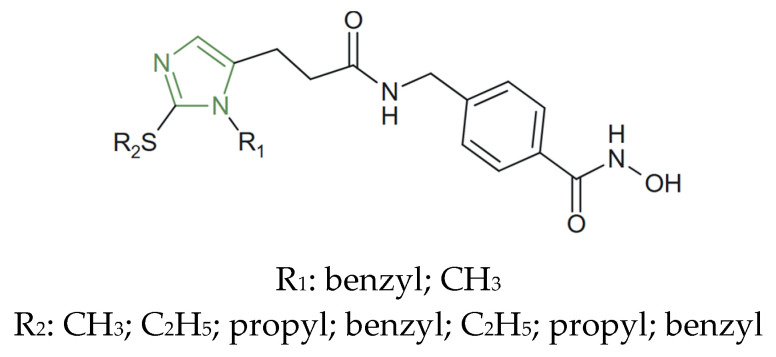
Chemical structure of new anticancer imidazole derivatives [62].

A series of new imidazole derivatives (Figure 28) were also designed and synthesized by Nasrollahzadeh et al. as histone deacetylase (HDAC) inhibitors, inspired by known imidazole derivatives as HDAC inhibitors and the tridimensional structures of the enzyme’s active site. They demonstrated potent HDAC inhibitory activity through a conserved binding mode involving coordination of the catalytic Zn^2+^ ion, hydrophobic interactions along the enzyme tunnel, stabilizing contacts at the pocket entrance which were comparable to entinostat, and exhibiting strong cytotoxic effects, especially analogues bearing a benzyl substituent on the imidazole ring and small alkyl groups, such as methyl, ethyl, or propyl on the amide side chain, which exhibited the most potent activities IC_50_ = 1.48–3.07 µM against HCT116. Molecular dynamics simulations (100 ns) confirmed that the new derivatives form stable complexes with HDAC1 through strong hydrophobic interactions, zinc ion coordination, and hydrogen bond formation, supporting its high inhibitory potency [62].

**Figure 29:**
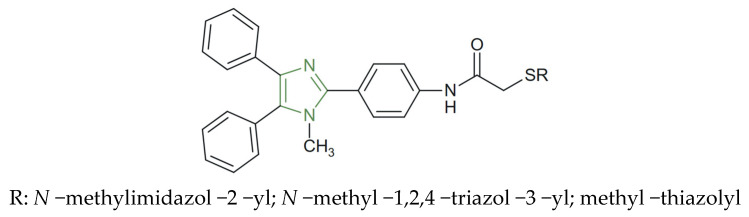
Chemical structure of anticancer triarylimidazole derivatives [142].

In another study, a series of a hybrid imidazole–imidazole/thiazole/1,2,4 -triazole/thiazolyl derivatives were synthesized and evaluated for their anticancer potential by Özkay et al. Preliminary in vitro studies on HT-29 and MCF-7 cell lines revealed that imidazole–(benz)azole derivatives, as represented in Figure 29, displayed superior anticancer activity compared to their imidazole–piperazine counterparts, with IC_50_ values ranging between 1.6–10.7 µg/mL on the HT-29 and 3.2–6.5 µg/mL on the MCF-7 cell line. Among the tested analogues, those with 1,2,4-triazole or 1,2,4-thiadiazole rings showed the highest selectivity against HT-29 colon carcinoma cells, outperforming the standard drug cisplatin IC_50_ = 1.7 µg/mL. These compounds induced DNA fragmentation but showed limited inhibition of DNA synthesis, suggesting that their anticancer mechanism acts primarily after the completion of cellular DNA synthesis [142].

**Figure 30:**
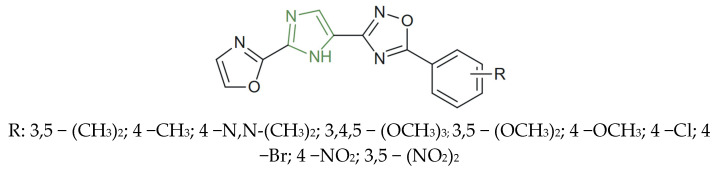
Chemical structure of new anticancer 1,2,4-oxazolyl–imidazole derivatives [143].

A new series of 1,2,4-oxadiazole-incorporated (2-(oxazol)-1*H*-imidazole) derivatives were synthesized by Bairi et al., as presented in Figure 30. The compounds were evaluated for their preliminary anticancer activity against four human cancer cell lines, including prostate cancer (PC3, DU-145), lung cancer (A549), and breast cancer (MCF-7), using etoposide as a positive control. The results demonstrated their excellent to moderate anticancer activity compared to the positive control [143].

**Figure 31:**
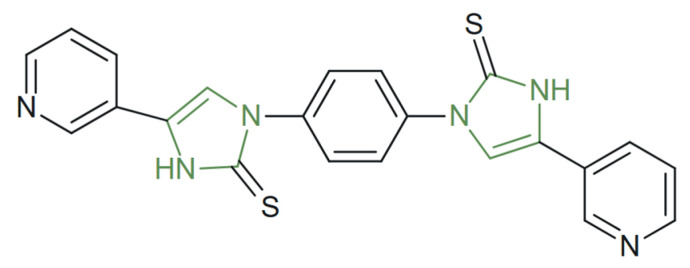
Chemical structure of novel anticancer imidazole–thione derivative [57].

In a recent study, Alzahrani generated a series of imidazole-2-one/-2-thione derivatives via an atom-economical multicomponent strategy, introducing heteroaryl fragments at C2 of the imidazole core (Figure 31) and pyridyl groups, respectively. An in vitro antiproliferative screening against HepG-2 and MCF-7 revealed that pyridyl-substituted imidazole-2-thiones displayed noteworthy cytotoxicity against HepG-2 IC_50_ = 13.48 ± 1.10 µM while showing lower potency toward MCF-7. Doxorubicin, used as the positive control, displayed IC_50_ = 4.12 ± 0.25 µM onHepG-2 cell line and IC_50_ = 5.27 ± 0.33 µM on MCF-7, confirming the strong but comparable activity of the tested derivative to the reference drug. The SAR thus supports imidazole-2-thione scaffolds bearing pyridyl groups as more efficacious analogues in this series [57].

**Figure 32:**
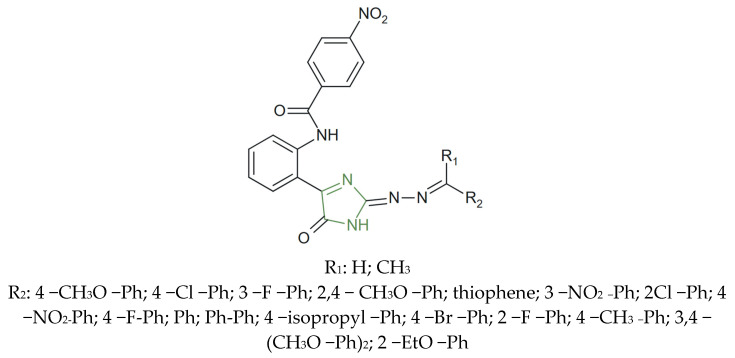
Chemical structure of new imidazole derivatives with anticancer activity [144].

In another study, Tapera et al. explored the potential of a series of imidazole-based derivatives incorporating a hydrazone moiety (as presented in Figure 32) against colorectal cancer cell lines HT-29 and HCT-116. Among the tested molecules, several compounds bearing electron-donating or electron-withdrawing groups on the aryl hydrazone segment exhibited strong cytotoxic effects [144].

SAR investigations underscore the pivotal contribution of the imidazole nucleus within benzimidazole-based scaffolds to their anticancer potential. Substitutions at the second position, particularly with aryl or heteroaryl moieties, have been shown to enhance cytotoxic activity, likely through improved molecular interactions with key oncogenic targets. The electron-rich imidazole ring facilitates crucial binding interactions, reinforcing its role as a core pharmacophore in the rational design of benzimidazole-derived anticancer agents.

**Figure 33:**
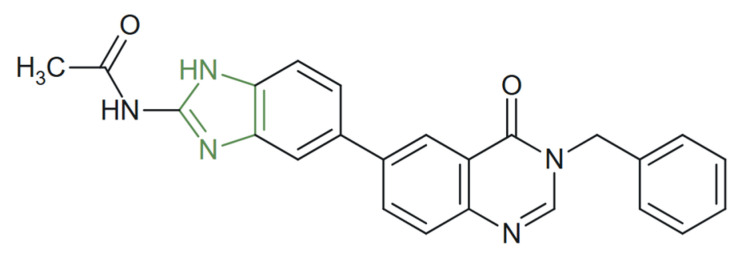
Chemical structure of novel anticancer benzimidazolyl−quinazolin-one derivatives [145].

For instance, Fan et al. synthesized a series of novel quinazolin-4(3*H*)-one derivatives containing imidazole moieties (Figure 33) which were evaluated as potential multi-targeted inhibitors of Aurora A, PI3K, and BRD4. Among these, one demonstrated superior antiproliferative activity against A549, HCC827, and H1975 cancer cell lines, also confirmed by biochemical assays, which potently inhibited Aurora A and PI3Kα kinase activities [145].

**Figure 34:**
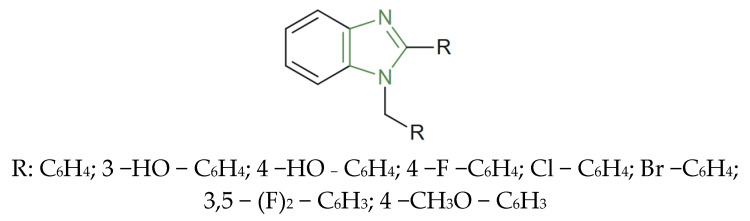
Chemical structure of new anticancer benzimidazole derivatives [146].

Avila-Sorrosa et al. developed a series of 1-benzyl-2-aryl disubstituted benzimidazole derivatives (Figure 34), and a Schiff base using a straightforward, environmentally friendly approach involving recyclable heterogeneous catalysts (zeolites) under mild reaction conditions. In vitro anticancer evaluations revealed significant bioactivity for halogenated derivatives; particularly, the chlorinated and brominated derivatives exhibited the highest cytotoxicity, reaching inhibition levels of 86–88% against leukemia and 78–86% against breast cancer cells, while fluorinated analogues showed moderate activity (30–50% inhibition) on HCT-15 and SKLU-1 cell lines, and the difluorinated variants retained a balanced activity across all tested cancers (≈25–45%). The methoxylated derivative showed potent effects—reaching inhibition levels of 45% against leukemia (K562) and up to 60% inhibition levels on lung (SKLU-1) cancer cell lines [146].

**Figure 35:**
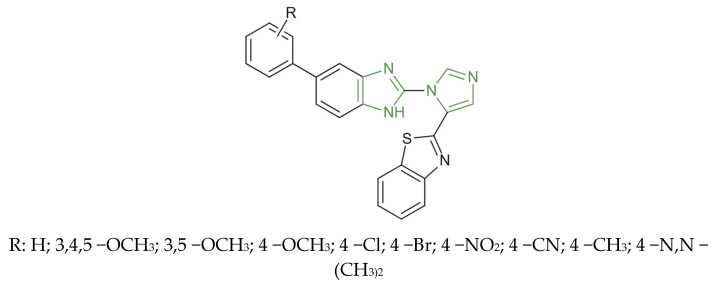
Chemical structure of novel anticancer benzimidazole–imidazolyl–benzothiazole derivatives [147].

A series of novel benzimidazole–imidazolyl–benzothiazole derivatives were synthesized by Edukondalu et al., as represented in Figure 35, which were evaluated for their anticancer potential against four human cancer cell lines: MCF-7 (breast), A549 (lung), Colo-205 (colon), and A2780 (ovarian). Among the evaluated derivatives, the methoxy-substituted analogues were particularly notable, exhibiting anticancer activity with IC_50_ values of 0.018 ± 0.003 µM (MCF-7), 0.011 ± 0.001 µM (A549), 0.012 ± 0.029 µM (Colo-205), and 0.17 ± 0.023 µM (A2780), surpassing the potency of the reference drug etoposide (IC_50_ ≈ 1–3 µM). Derivatives containing two methoxy groups at 3,5-positions or a single 4-methoxy group showed moderate activity (IC_50_ = 1.22–1.88 µM), while those bearing electron-withdrawing substituents, such as –Cl, –Br, –CN, or –NO_2_, exhibited markedly reduced cytotoxic effects (IC_50_ > 5 µM) [147].

**Figure 36:**
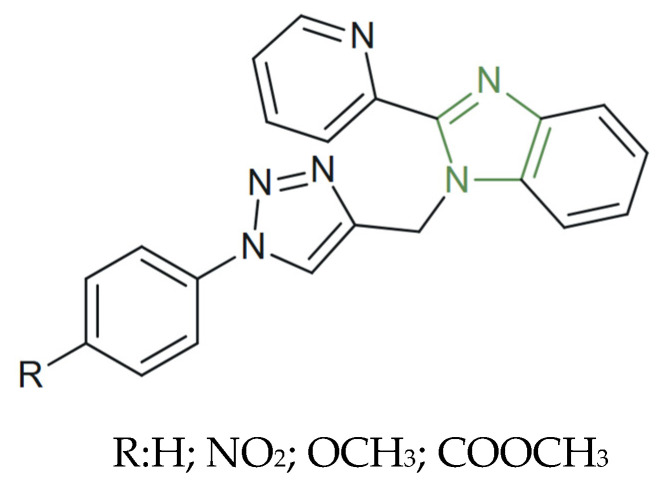
Chemical structure of novel anticancer 1,2,3-triazole benzimidazoles derivatives [58].

Finally, among the studies we analyzed, there is also a series of 1,2,3-triazole-pyridine-benzo[d]imidazole derivatives (Figure 36) developed and presented by Al-Qahtani et al. Their cytotoxic potential was investigated through an LDH assay against three human cancer cell lines (HepG2, MCF-7, and HCT116) and a normal human fibroblast cell line (BJ-1) using doxorubicin as a reference. Among the tested analogues, the triazole derivative containing a pyridine bridge and an unsubstituted benzimidazole nucleus showed the strongest inhibition on liver (HepG2, IC_50_ = 8.0 ± 0.2 µM) and breast (MCF-7, IC_50_ = 3.2 ± 0.1 µM) cancer cells, while maintaining moderate selectivity toward colon carcinoma (HCT116, IC_50_ = 5.7 ± 0.2 µM). The results revealed a dose-dependent cytotoxic effect across all cancer cell lines, with higher cytotoxicity against colorectal cancer cells (HCT116) than doxorubicin, indicating potency and selectivity in this cell line. Molecular docking studies supported the observed bioactivity, highlighting the favorable ligand–protein interactions likely contributing to their cytotoxic mechanisms [58].

The collected studies demonstrate the remarkable versatility of the imidazole scaffold in anticancer drug development, as subtle structural variations are consistently translated into pronounced differences in cytotoxic potency and selectivity across multiple cancer models. For instance, compounds incorporating electron-donating substituents, particularly methoxy- or trimethoxy-substituted aromatic systems, frequently exhibited superior antiproliferative activity, in some cases reaching nanomolar potency and outperforming established reference drugs [147]. Likewise, small alkyl or thioalkyl substitutions on the imidazole nucleus were repeatedly associated with enhanced cytotoxic effects, especially in imidazole–chalcone and phenylahistin-derived systems.

Hybridization of the imidazole core with other heterocycles, including oxadiazoles, triazoles, benzothiazoles, or pyrroles, further expanded the anticancer potential of these compounds, frequently yielding molecules capable of interacting with multiple oncogenic targets and demonstrating improved efficacy against resistant cancer cell lines.

## 3. Conclusions

Imidazole derivatives constitute one of the most intensively studied classes of heterocyclic compounds in contemporary medicinal chemistry, largely due to their unique ability to serve as bioisosteres for various pharmacophores and to modulate crucial biochemical pathways, while the structural framework of the imidazole ring—aromatic, amphoteric, and highly polar—confers both chemical versatility and biological functionality, making it a privileged scaffold in the rational design of pharmacologically active compounds.

Considering their wide-ranging therapeutic applications—from antifungal and antibacterial to anticancer, antitubercular, and CNS-related disorders—imidazole derivatives remain at the forefront of modern medicinal chemistry, as their synthetic accessibility, chemical adaptability, and complex mechanisms of action render them highly promising scaffolds for future pharmacological innovation, with the potential to yield safer, more selective, and more effective therapeutic agents.

Numerous studies presented in this paper have showed that imidazole moieties containing molecules possess a wide potential for current use as antifungal, antibacterial, anti-inflammatory, anti-ulcer, and anticancer therapeutic compounds, among other uses. In line with modern drug development paradigms, structure–activity relationship (SAR) and quantitative structure–activity relationship (QSAR) analyses have revealed that substitutions at the N1, C2, and C5 positions of the imidazole ring and, more generally, covalent engagement of the imidazole core at the N1, C2, and C5 positions, have a major impact on the biological potency and selectivity of imidazole derivatives, as demonstrated by the extensive in vitro evaluations reported in this article. The imidazole nucleus does not merely function as a simple heterocycle, but rather acts as a central structural element, being covalently connected mainly through the N1, C2, and C5 positions, which significantly influences molecular topology, electronic distribution, and the spatial orientation of the associated pharmacophoric fragments.

Engagement at the N1 position contributes to enhanced biological activity, particularly in antifungal, anticancer, and antimicrobial applications, where N1-involved derivatives exhibit increased potency associated with improved lipophilicity. This effect is especially pronounced when N1 engagement is combined with electron-withdrawing substituents, which enhance antibacterial activity by modulating the electronic properties of the scaffold and facilitate interactions with bacterial membranes and intracellular targets.

Engagement of the C2 position also plays an important role in modulating biological activity, as covalent attachment or substitution at this position—particularly with electron-withdrawing groups—has been associated with enhanced antitumor, antibacterial, and antidiabetic effects, including superior α-glucosidase inhibition compared to acarbose.

Furthermore, covalent connections through the C5 position emerge as the most biologically relevant connection, as demonstrated by the prominent role of 5-nitroimidazole derivatives, where the presence of a strongly electron-withdrawing nitro group at C5 is essential for biological activity, enabling a well-established redox-selective activation mechanism responsible for antiparasitic, antibacterial, and antitumor effects.

Future investigations into imidazole-based scaffolds are anticipated to advance along several critical trajectories as computational chemistry represents an important and complementary tool in the discovery and optimization of new imidazole derivatives. A significant proportion of the studies cited in this work are supported by structure–activity relationship (SAR) analyses which consistently demonstrate the positive impact of introducing specific substituents—particularly electron-withdrawing groups—in biological activity, providing a rational starting point for the further optimization of imidazole-based scaffolds and the design of new analogues with improved pharmacological profiles. The integration of computational methodologies, including molecular docking, molecular dynamics simulations, and artificial intelligence-driven QSAR modeling, is expected to accelerate the identification and optimization of novel imidazole derivatives with improved pharmacological efficacy and safety margins, as molecular docking studies enable the theoretical evaluation of the binding affinity and selectivity of newly designed compounds toward relevant biological targets prior to synthesis, thereby allowing the prioritization of the most promising candidates for experimental validation. Further, the integration of computational methods facilitates informed decision-making in lead selection, reduces unnecessary synthetic efforts and, in the long term, contributes to improved experimental sustainability throughout the drug discovery process. Such in silico strategies may also enable the early prediction of selectivity, toxicity, and pharmacokinetic behavior, thereby facilitating a more informed selection of lead candidates prior to experimental validation. In parallel, the rational design of hybrid scaffolds, in which the imidazole nucleus is fused or conjugated with complementary pharmacophores, holds considerable promise for the development of multifunctional therapeutics targeting complex and multifactorial diseases such as cancer, diabetes, and neurodegenerative disorders. Hybridization strategies build directly on SAR- and docking-guided insights, enabling the selection of optimal substitution patterns and pharmacophoric combinations prior to synthesis, while this may further contribute to overcoming drug resistance and enhancing therapeutic durability by simultaneously modulating multiple biological targets. Additionally, the implementation of sustainable synthetic approaches, for example microwave-assisted, solvent-free, and catalytic methodologies, will not only improve synthetic efficiency and yields, but align with the principles of green chemistry, thereby reducing the environmental impact of large-scale production.

## Data Availability

No new data were created or analyzed in this study. Data sharing is not applicable to this article.

## Abbreviations

The following abbreviations are used in this manuscript:

A2780	Human ovarian cancer cell line
A549	Human alveolar basal epithelial adenocarcinoma cell line
ADME	Absorption, Distribution, Metabolism, Excretion
ATP	Adenosine triphosphate
BCL-2	B-cell lymphoma 2
BJ-1	Normal human fibroblast cell line
BRD4	Bromodomain-containing protein 4
BTK	Bruton’s tyrosine kinase
Colo-205	Human colorectal adenocarcinoma cell line
DDP	Cisplatin-resistant
DMAD	Dimethyl acetylenedicarboxylate
DNA	Deoxyribonucleic acid
DU-145	Human prostate carcinoma cell line
G2/M	G2/M phase of the cell cycle
GABA	Gamma-aminobutyric acid
H1975	Human lung adenocarcinoma cell line
H357	Human oral squamous carcinoma cell line
H37 Rv	Standard references for *Mycobacterium tuberculosis*
HCC827	Human non-small cell lung cancer cell line
HCT116	Human colorectal carcinoma cell line
HCT-15	Human colorectal adenocarcinoma cell line
HDAC	Histone deacetylase
HEK	Human embryonic kidney cells
HeLa	Human cervical cancer cell line
Hep G2	Hepatocellular carcinoma
HOMO	Highest occupied molecular orbital
HT-29	Human colorectal adenocarcinoma cell line
IC_50_	Inhibitory concentration 50%
IR	Infrared Spectroscopy
K562	Human chronic myelogenous leukemia cell line
LDH	Lactate dehydrogenase
LUMO	Lowest occupied molecular orbital
MCF-7	Human breast cancer cell line
MD	Molecular dynamics
MDA-MB-231	Triple-negative human breast cancer cell line
MELK	Maternal embryonic leucine zipper kinase
MIA Pa Ca-2	Human pancreatic adenocarcinoma cell line
MIC_50_	Minimum inhibitory concentration 50%
MM/GBSA	Molecular mechanics/Generalized born surface area
MTT	3-(4,5-dimethylthiazol-2-yl)-2,5-diphenyltetrazolium bromide
NMR	Nuclear magnetic resonance
P	Perimeter
PC3	Human prostate cancer cell line
PI3K	Phosphatidylinositol 3-kinase
PP2A	Protein phosphatase 2A
QSAR	Quantitative structure–activity relationship
Rac1	Ras-related C3 botulinum toxin substrate
RAW	Murine macrophage cell line
RI	Resistance index
RNA	Ribonucleic acid
SAR	Structure–activity relationship
SKLU-1	Human lung carcinoma cell line
SYK	Spleen tyrosine kinase
TBTA	Tris(benzyltriazolylmethyl)amine
TCD50	Tumor control dose 50%
